# Using purine skews to predict genes in AT-rich poxviruses

**DOI:** 10.1186/1471-2164-6-22

**Published:** 2005-02-18

**Authors:** Melissa Da Silva, Chris Upton

**Affiliations:** 1 Department of Biochemistry and Microbiology, University of Victoria, Victoria, BC, V8W 3P6, Canada

## Abstract

**Background:**

Clusters or runs of purines on the mRNA synonymous strand have been found in many different organisms including orthopoxviruses. The purine bias that is exhibited by these clusters can be observed using a purine skew and in the case of poxviruses, these skews can be used to help determine the coding strand of a particular segment of the genome. Combined with previous findings that minor ORFs have lower than average aspartate and glutamate composition and higher than average serine composition, purine content can be used to predict the likelihood of a poxvirus ORF being a "real gene".

**Results:**

Using purine skews and a "quality" measure designed to incorporate previous findings about minor ORFs, we have found that in our training case (vaccinia virus strain Copenhagen), 59 of 65 minor (small and unlikely to be a real genes) ORFs were correctly classified as being minor. Of the 201 major (large and likely to be real genes) vaccinia ORFs, 192 were correctly classified as being major. Performing a similar analysis with the entomopoxvirus amsacta moorei (AMEV), it was found that 4 major ORFs were incorrectly classified as minor and 9 minor ORFs were incorrectly classified as major. The purine abundance observed for major ORFs in vaccinia virus was found to stem primarily from the first codon position with both the second and third codon positions containing roughly equal amounts of purines and pyrimidines.

**Conclusion:**

Purine skews and a "quality" measure can be used to predict functional ORFs and purine skews in particular can be used to determine which of two overlapping ORFs is most likely to be the real gene if neither of the two ORFs has orthologs in other poxviruses.

## Background

In 1966, Szybalski first discovered that the mRNA synonymous strand of DNA contained a predominance of purine-rich clusters [1]; by convention, the top strand of a linear dsDNA molecule is viewed 5'→3', therefore when transcription of a gene is to the right, the top strand is considered the mRNA synonymous strand and if transcription is to the left, the top strand is the template strand. Chargaff's second parity rule states that for single-stranded DNA %A ≅ %T and %C ≅ %G [2,3] and implies that for regions with clusters of purines there must be local deviations from Chargaff's second parity rule favoring purines [4]. These local deviations from Chargaff's second parity rule also known as Chargaff differences have been seen in a variety of organisms including vaccinia virus; Bell *et al. *determined that Chargaff differences do correlate with direction of transcription and that the number of A nucleotides is greater than the number of T nucleotides in 83 of 92 vaccinia genes [4].

Many programs have been designed to predict genes, but few actually rate the "quality" or significance of the prediction and leave researchers to evaluate this themselves. In poxviruses, predicting which ORFs are likely to be expressed (genes) without the use of biochemical analysis usually involves simply choosing a minimum ORF length cut-off and excluding all ORFs that are smaller than the cut-off. Analysis may be extended to include manual inspection of each predicted ORF for the presence of promoter consensus sequences. Excluding ORFs that are smaller in size than the cut-off, however, risks missing genes that are unusually short; during annotation of vaccinia virus strain Copenhagen (VACV-COP) at least three recently verified genes (ranging from 162 bp – 231 bp) were not included in the initial annotation of the complete genome; these genes, VACV-COP A2.5L [5,6], A14.5L [7] and G5.5R [8] have now been included in our Poxvirus Orthologous Clusters (POCs) database [9].

Poxvirus genes are transcribed from both DNA strands and so far have never been shown to overlap more than a few nucleotides. Despite this knowledge, some poxvirus genomes have been liberally annotated so as to include all ORFs above a certain size, irrespective of whether they overlap larger well-characterized genes. Thus, the current GenBank file for VACV-COP contains 202 major (large and likely to be real genes) ORFs and 64 minor (small and unlikely to be real genes) ORFs [10,11]. The majority of these minor ORFs in VACV-COP overlap larger, major ORFs on the opposite DNA strand.

In this paper, it is shown that for the AT rich poxviruses, the purine skews can be used to help predict the synonymous (coding) strand, particularly in regions where smaller ORFs overlap each other on opposite strands of the genome and neither have orthologs in other poxvirus genomes. Furthermore, it is shown that the majority of minor ORFs found in VACV-COP are unlikely to be functional genes and that based on purine content, two of the three genes initially excluded from the annotation of the vaccinia virus genome due to their small size, fit our definition of a major ORF.

## Results and discussion

**Figure 1:**
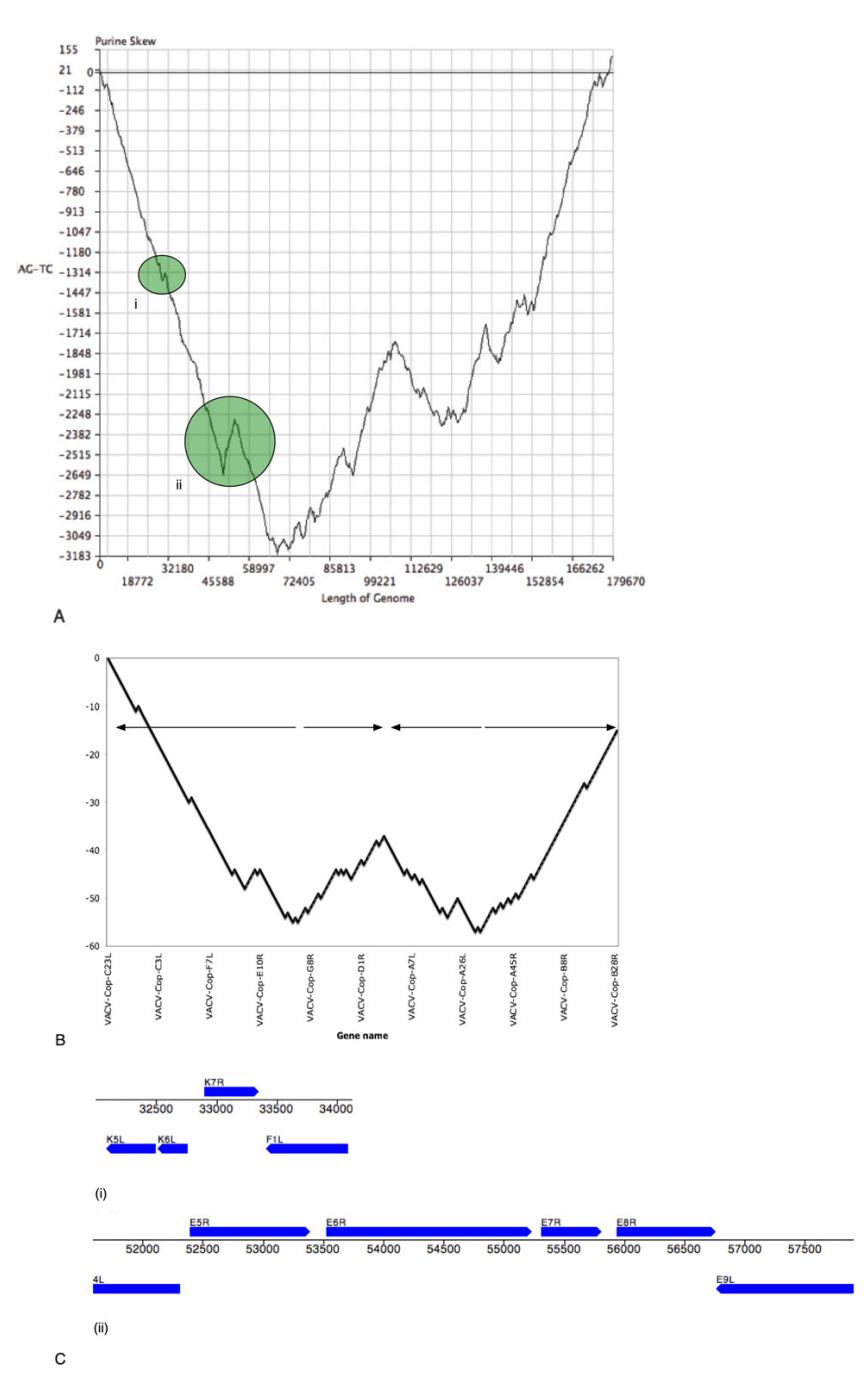
Correlation between purine skew and direction of transcription of VACV-COP genome, excluding the non-coding terminal inverted repeats. (a) Purine skew drawn using DNAGrapher. Regions of the top strand that exhibit a purine bias will have a trend to the upward direction whereas regions that exhibit a pyrimidine bias will be drawn in the downward direction. Two example regions of changes in strand bias are shaded in green and marked (i) and (ii) (b) VACV-COP major ORFs drawn according to the strand of the genome on which each ORF is located. Beginning with a value of zero for the first major ORF of the genome, a numerical value of +1 or -1 is added to the value of the previous ORF depending on if the ORF is located on the top or bottom strand, respectively. (c) Gene orientation in two example regions demonstrating a change in strand bias. (i) Strand bias changes from a purine bias on the bottom strand, to a purine bias on the top strand that encompasses 1 gene on the top strand. (ii) Strand bias changes from a purine bias on the bottom strand, to a purine bias on the top strand that encompasses 4 genes located on the top strand.

Figure 1 shows the genomic purine skew (Figure 1a) and the direction of transcription (Figure 1b) for the major ORFs (genes) in VACV-COP. Since the major ORFs of VACV-COP are spread out evenly across the genome, and Figure 1b was created using only the major VACV-COP ORFs, the two figures (Figure 1a and 1b) follow very similar trends. A characteristic "W" shaped plot can be seen for both graphs; in Figure 1b, this is the result of a trend for large blocks of genes to be transcribed in the same direction (see arrows in Figure 1b). These data indicate a good correlation between the purine content of the genomic DNA and the direction of transcription; for example, for genes that are transcribed in the leftward direction, the bottom/synonymous strand is purine rich and the opposite is true for genes that are transcribed to the right. The correlation between purine content and the likelihood that an ORF is major is further supported by the fact that 180 of the 202 major ORFs of VACV-COP have a purine content greater than or equal to 50%. In this way, purine skews can be used to help annotate newly sequenced genomes by aiding in the determination of the mRNA synonymous strand.

When the purine skew (Figure 1a) slopes in the downward direction, this is due to a pyrimidine bias on the top strand and a commensurate purine bias on the bottom strand indicating that the major ORFs are located on the bottom strand. In regions where the purine skew changes direction from a downward slope to an upward slope or vice versa, these are regions on the genome where the transcription direction of the genes in the genome changes. For example, the purine skew appears to change direction from a downward slope to an upward slope at position 32,800 bp and then changes again from an upward slope to a downward slope at position 33,500. Figure 1c (i) shows that within this region (32,800–33,500 bp), there is one gene (VACV-COP K7R) that is located on the top strand (upwards slope on purine skew) and is flanked by genes that are located on the bottom strand (downward slope on purine skew). A second example can be seen in figure 1 c (ii) where an upward slope in the purine skew occurs between positions 52,400 and 57,000. In this case, the upward sloping region encompasses four genes (VACV-COP E5R, E6R, E7R and E8R) and the two downward sloping regions flanking each side of this region encompass genes that are located on the bottom strand.

**Figure 2:**
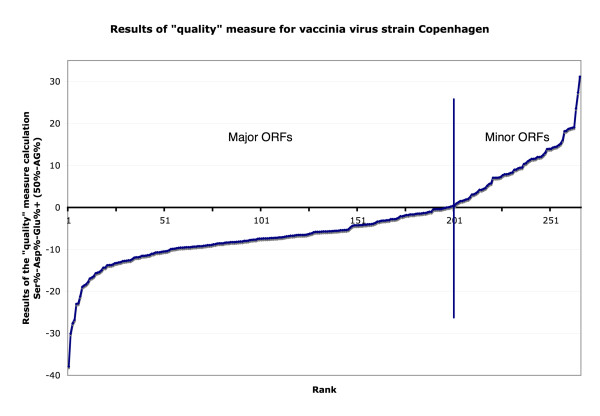
Results of the "quality" measure for VACV-COP. Y-axis plots results of the "quality" calculation (Ser%-Asp%-Glu%+[50%-AG%]) and X-axis depicts rank of each ORF.

It was previously shown that minor ORFs in VACV-COP tend to have higher than average serine content as well as lower than average aspartate and glutamate content [12]. Based on these observations and our current finding that the synonymous DNA strand is usually purine rich, we created a simple mathematical equation designed to provide a "quality" measure of each ORF. The results of the formula [Ser%-Asp%-Glu%+(50-AG%)], which essentially sums the trends in amino acid composition (3 amino acids) and purine content, are shown in Figure 2. If peptides are translated from ORFs on the non-synonymous strand, they tend to have a higher than average Ser%, but lower than average Asp% and Glu% (due to properties of the genetic code), and have a lower than average purine content. By subtracting the actual %purine from the genome average for VACV-COP (50%), if the ORF is major, the numerical result of the equation is negative and if the results of the equation are positive, the ORF is predicted to be minor.

Plotting the results of this equation, we found that of the 266 ORFs originally predicted in VACV-COP, 6 ORFs (VACV-COP A ORF G, VACV-COP A ORF T, VACV-COP B ORF G, VACV-COP C ORF F, VACV-COP E ORF D, and VACV-COP F ORF A) were incorrectly classified as being major and 9 ORFs (VACV-COP A9L, VACV-COP A13L, VACV-COP A14L, VACV-COP A14.5L, VACV-COP A38L, VACV-COP A43R, VACV-COP C3L, VACV-COP I5L, VACV-COP I6L) were incorrectly classified as being minor.

It was found that the majority of incorrectly classified major ORFs were misclassified because they are small membrane proteins that had a lower aspartate and glutamate content than other major ORFs and that the majority of incorrectly classified minor ORFs were misclassified because they have a lower serine and higher purine percentage compared to other minor ORFs despite the fact that all but one minor ORF (VACV-COP A ORF T) overlap a major ORF on the opposite strand (Table 1). There were three genes that had initially been excluded from the annotation of VACV-COP due to their small size. Two of these genes (VACV-COP A2.5L and VACV-COP G5.5R) have a negative "quality" measure value indicating that they are major. One of these genes (VACV-COP A14.5L) was misclassified as minor likely due to the fact that it is a small membrane protein (Table 1).

**Table 1:** List of VACV-COP ORFs that were incorrectly classified.

**Major ORFS incorrectly classified as minor**						
ORF name	ORF size (bp)	Serine content (%)	Aspartate content (%)	Glutamate content (%)	Purine content (%)	Explanation

VACV-COP A13L	210	11.43	1.43	2.86	48.82	Small, membrane protein
VACV-COP A14L	270	11.11	3.33	0	45.79	Small, membrane protein
VACV-COP A14.5L	159	7.55	1.89	1.89	44.45	Small, membrane protein
VACV-COP A38L	831	7.94	3.97	2.53	47.25	Membrane protein
VACV-COP A43R	582	10.31	5.67	1.55	51.11	Membrane protein
VACV-COP C3L	789	13.31	4.18	3.8	52.27	High Ser%, low Asp% and Glu%
VACV-COP I5L	237	5.06	2.53	1.27	49.58	Small, membrane protein
VACV-COP I6L	1146	10.99	4.45	4.45	49.7	High Ser%, low Asp% and Glu%

**Minor ORFs incorrectly classified as major**						

ORF name	ORF size (bp)	Serine content (%)	Aspartate content (%)	Glutamate content (%)	Purine content (%)	Explanation

VACV-COP A ORF G	225	6.67	4	8	54.39	Low Ser%, high Asp% and AG%
VACV-COP A ORF T	243	1.23	3.7	2.47	51.63	Overlaps on same strand as major ORF
VACV-COP B ORF G	273	1.1	3.3	1.1	53.26	Low Ser%, high AG%
VACV-COP C ORF F	273	1.1	3.3	1.1	53.26	Low Ser%, high AG%
VACV-COP E ORF D	198	9.09	4.55	6.06	55.72	High Asp%, Glu%, AG%
VACV-COP F ORF A	201	4.48	4.48	0	50.49	Low Ser%

**Figure 3:**
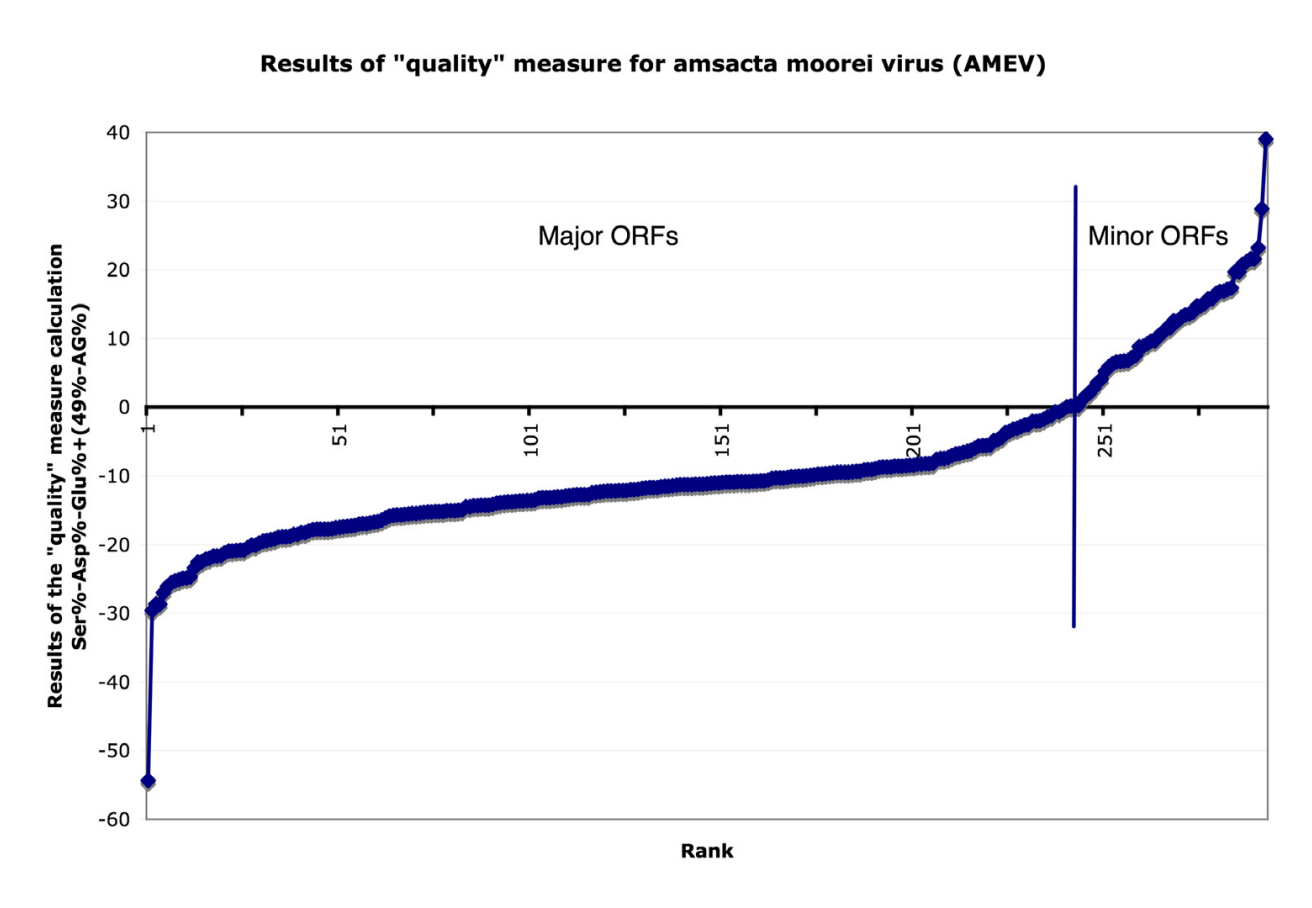
Results of the "quality" measure for amsacta moorei virus (AMEV). Y-axis plots results of the "quality" calculation (Ser%-Asp%-Glu%+[49%-AG%]) and X-axis depicts rank of each ORF.

A similar analysis was repeated for the genome of amsacta moorei (AMEV), an extremely AT-rich (82%) entomopoxvirus [13]. The AMEV genome was chosen for two reasons: (1) because it is not closely related to any known poxviruses and therefore its genome contains a large number of genes with unknown function and (2) its genome was liberally annotated and therefore it is questionable which ORFs are likely to be functional genes. Thus, the "quality" measure was used to predict which AMEV ORFs are most likely to be minor. Figure 3 graphically depicts the results of the "quality" measure calculation for AMEV. Due to the extreme AT-richness of the AMEV genome, it was necessary to modify the "quality" measure to the following formula: [Ser%-Asp%-Glu%+(49%-AG%)]. 49% was chosen instead of 50% for the purine portion of this equation since the average purine content of the entire AMEV genome is 49%. As was the case with VACV-COP, if the ORF is minor, the results of the "quality" measure will be positive.

It was found that there were 51 ORFs that had a positive "quality" value and are therefore considered minor. Of these 51 ORFs, 41 ORFs further fit our definition of a minor ORF as they overlapped another larger ORF on the opposite strand and 4 major ORFs (AMEV-161, AMEV-164, AMEV-171, and AMEV-183) were incorrectly classified as minor even though they each have orthologs in other poxviruses and are therefore major (Table 2). The remaining 6 ORFs (AMEV-001, AMEV-089, AMEV-148, AMEV-198, AMEV-ITR02, and AMEV-ITR08) that were classified as minor using our "quality" measure were found not to overlap any ORFs on the opposite or same DNA strand and were further analyzed using the AMEV purine skew in order to try and determine the correct coding strand in each of these 6 regions (Table 3). It was found that for 5 (AMEV-001, AMEV-089, AMEV-148, AMEV-198, AMEV-ITR02) of these 6 ORFs, the purine skew indicates a coding strand opposite the strand on which these ORFs are located, or in other words, that these ORFs are minor. For 1 (AMEV-ITR08) ORF, the purine skew indicated a coding strand identical to the strand on which this ORF is located and therefore this ORF may actually be major. AMEV-ITR08 does not have any orthologs in other poxviruses but it does show a 73.6% amino acid identity with the AMEV-ITR07 ORF which was classified as being major using the "quality" calculation further supporting that AMEV-ITR08 is likely major. AMEV-ITR08 was predicted to contain a transmembrane domain [13] which could explain why it was misclassified.

**Table 2:** List of AMEV ORFs that were incorrectly classified.

**Major ORFS incorrectly classified as minor**						
ORF name	ORF size (bp)	Serine content (%)	Aspartate content (%)	Glutamate content (%)	Purine content (%)	Explanation

AMEV-161	243	11.11	2.47	1.23	47.56	Membrane protein
AMEV-164	708	7.63	2.12	2.97	47.97	High Ser%, low Asp% and Glu%
AMEV-171	276	3.26	1.09	1.09	48.39	Low Asp% and Glu%
AMEV-183	675	6.67	3.11	1.33	51.18	Low AG% and low Glu%

**Minor ORFs incorrectly classified as major**						

ORF name	ORF size (bp)	Serine content (%)	Aspartate content (%)	Glutamate content (%)	Purine content (%)	Explanation

AMEV-152	225	0	12	1.33	60.97	Overlaps on same strand as major ORF
AMEV-189	180	1.67	8.33	1.67	43.17	Low Ser%, high Asp%
AMEV-191	228	0	2.63	10.53	61.9	Overlaps on same strand as major ORF

**Table 3:** List of 6 AMEV ORFs classified as minor that do not fit the definition of a minor ORF and conclusions as to whether or not they are minor.

**ORF name**	**DNA strand on which ORF is located**	**Direction of purine skew**	**Conclusion**
AMEV-001	Top	Down	Minor
AMEV-089	Top	Down	Minor
AMEV-148	Bottom	Up	Minor
AMEV-198	Bottom	Up	Minor
AMEV-ITR02	Top	Down	Minor
AMEV-ITR08	Top	Up	May be major

There were three ORFs that had been classified as major (negative value for the "quality" measure) yet overlapped a larger gene on the opposite or same DNA strand (Table 2). Two of these ORFs (AMEV-152 and AMEV-191) overlap a larger ORF on the same strand and therefore neither the purine skew nor the "quality" measure are capable of determining which ORF is major; and one ORF (AMEV-189) overlaps the much larger spheroidin gene on the opposite strand and was likely misclassified due to its lower than average serine content and higher than average aspartate content.

For the analyses shown in figures 2 and 3, the cut-off value used in both cases was zero. The value of zero was chosen in the training case (VACV-COP) because it represented a reasonable cut-off between genes that were known to be major and ORFs that were known to be minor with minimal misclassification of genes. With our test case (AMEV), since it was not known which ORFs were major or minor, a cut-off of zero was initially used with the presumption that the cut-off may need to be adjusted due to the extreme AT-richness of the AMEV genome. Analyzing the "quality" measure data obtained for AMEV with a cut-off of zero yielded satisfactory results in that the number of overlapping and therefore likely to be minor ORFs that were misclassified was relatively low and because of this we decided to maintain the zero cut-off. It is likely that a cut-off of zero worked well with AMEV despite its extremely AT-rich genome because the "quality" measure that was used reflected the average AG% of the genome. It is also likely that other poxvirus genomes that are analyzed using our method would use a cut-off of zero, provided the "quality" measure that was used was changed to reflect the average AG content of the genome, although we have yet to test whether this cut-off is universal throughout all poxviruses.

Thus far we have shown that purine skews can be used to predict the coding strand of poxvirus genomes and that major ORFs in VACV-COP and in AMEV usually contain greater than 50% and 49% purines, respectively. In order to explain this purine richness in genes, the purine (R) to pyrimidine (Y) ratio (R:Y) was calculated for each codon position of each coding and non-coding ORF in VACV-COP. A Student's T-test was used to compare the mean R/Y ratio values for the coding (genes) and non-coding ORFs at each codon position; means were considered statistically different when the p-value was less than 0.05. At the first nucleotide position in the codon, both VACV-COP major and minor ORFs are rich in purines but the major ORFs (genes) have significantly (p < 0.05) higher levels of purines at this position (Table 4). At the second nucleotide position the major ORFs have a R:Y ratio of approximately 1 and the minor ORFs have a significantly lower R:Y ratio (p < 0.05) indicating that minor ORFs are pyrimidine rich at the second codon position whereas major ORFs contain roughly equal amounts of purines and pyrimidines at this position. At position 3, no statistical difference was found, with both major and minor ORFs being rich in pyrimidines. Thus, for the first and second nucleotide positions of the codons, the major ORFs (genes) have significantly higher purine content than the minor ORFs.

**Table 4:** Mean purine to pyrimidine ratios for each codon position of vaccinia virus Copenhagen major and minor ORFs. Positions marked with an asterisk (*) are statistically different.

	Purine/Pyrimidine (R/Y) ratio at each codon position		
	
	Position 1*	Position 2*	**Position 3**
Major ORFs	1.77	0.99	0.93
Minor ORFs	1.21	0.75	0.96

It is important to remember that the use of purine/amino acid content of the coding strand and predicted protein, respectively, are just two measures that can be used to help predict whether an ORF is likely to be a functional gene and that usually they are only useful in discriminating between coding and non-coding strands. Occasionally ORFs that are fragments of *bone fide *genes are also flagged as non-functional, this is probably because of unusual amino acid content in small protein sub-domains. An example of this is the A25L ORF of VACV-COP that was flagged as non-functional by this method even though it is a fragment of the ATI protein. In a similar way, fragmentation of genes into smaller ORFs can also lead to unusual isoelectric points in the resulting predicted proteins; the 14 ORFs with a predicted pI of >9.6 are all minor ORFs or gene fragments. Thus, multiple approaches that may also include promoter analysis must be applied to attempt to correctly annotate small orphan ORFs in these genomes and there is no guarantee that the process will be 100% successful.

## Conclusion

We have successfully shown that in the case of AT-rich poxviruses, purine skews can be used to help predict the coding regions of the genome. This is particularly useful if predicted ORFs overlap each other and it is not apparently obvious which ORF is major (when neither ORF has an ortholog in another poxvirus genome). A second method that can be used in conjunction with purine skews is to calculate the "quality" of each predicted ORF using information from amino acid composition and purine content. For a given ORF, if the results of this calculation are negative the ORF is predicted to be a functional gene, and if the results of the calculation are positive, the ORF is predicted to be minor.

By comparing purine to pyrimidine (R/Y) ratios at each codon position of major and minor vaccinia virus ORFs, it was found that the purine abundance seen for major ORFs stems primarily from the first codon position with both the second and third codon positions containing equal amounts of purines and pyrimidines.

The software used to create the purine skews (DNAGrapher) and the VOCs database are both available for public use via the web [14,15].

## Methods

### Purine skews

Purine skews were created using the DNA Grapher feature in VOCs [9]. The DNA Grapher program implements the algorithm originally developed by Lobry [16]. The algorithm assigns a direction to each base encountered in the sequence. In the case of purine skews, the graph begins at position (0,0) and move upwards one unit if the base encountered is a purine (A or G) and moves downwards one unit if the base encountered is a pyrimidine, (C or T). The plot continues in this fashion until the end of the sequence is reached. A variable window size can also be set. In this case, the plot trend will be either upwards or downwards, depending on the average number of purines or pyrimidines in the window. The window then slides over the number of bases defined by the window size. For example, if the window size was defined as 10 bp, the window will slide over to the eleventh base and then count the average. The DNAGrapher program is integrated into the VOCs software and is also accessible as a Java WebStart program [14].

### Graphing ORFs by strand

The 202 major ORFs (genes) of VACV-COP were ordered in ascending order according to their start positions on the genome and then plotted according to which strand they are located using Microsoft Excel. The first gene was plotted at position 0 of the y-axis of the graph and a value of either -1 or +1 was added to the next gene on the genome depending on if it was on the bottom or top strand respectively.

### ORF "quality" calculation

The analysis of VACV-COP ORFs was performed by plotting the results of the following equation: Ser%-Asp%-Glu%+(50%-AG%) where Ser% is serine percentage, Asp% is aspartate percentage, Glu% is glutamate percentage, AG% is purine percentage and the value of 50% is the average purine content of the VACV-COP genome. The "quality measure" for AMEV ORFs used the following formula: Ser%-Asp%-Glu%+(49%-AG%) where the only modification of this formula from VACV-COP was the value of 49% which reflects the average purine content of the AMEV genome. The amino acid composition and purine data was obtained from the VOCs database which is available on the internet as a Java Web Start program [9,15].

The results of the equation for each ORF were tabulated, sorted in ascending order and assigned a rank from 1 being the ORF with the most negative value to either 266 in the case of VACV-COP or 292 in the case of AMEV being the ORF with the most positive value. These results of the calculation were plotted on the y-axis and the rank of each ORF was plotted on the x-axis using Microsoft Excel.

### Purine/pyrimidine ratio comparison

To analyse the ratio of purines to pyrimidines at each codon position, the total number of each nucleotide at each codon position was first calculated using the codontree program with the BC=A option (calculate the base composition at all 3 codon positions) selected [17,18]. Once the base composition at each codon position was calculated, the purine to pyrimidine ratio (R/Y) was calculated for each ORF of the dataset. The mean values of the R/Y ratio for each dataset were compared using Student's T-Test to determine if the mean R/Y ratio for each dataset was statistically different. The null hypothesis for the Student's T-test was that the means were equal and the null hypothesis was rejected if the p-value was < 0.05. The two datasets used for this portion of the paper consisted of (1) all ORFs classified as major in VACV-COP and (2) all ORFs classified as minor in VACV-COP.

## Authors' contributions

MD performed all analyses and wrote the manuscript. CU conceived of, and supervised the study, and edited the manuscript.
